# 

**Published:** 1884-08

**Authors:** 


ORIGINAL COMMUNICATIONS:

Tin and Gold Combined as a Filling Material Electrically and Practically Considered. W. D. Miller.......... 403
Treatment of Diseases of the Mouth. Julien W. Russell.......... 408
Dental Education—Rejoinder. Gorgas.......... 414
Dental Education— " Hopkinson.......... 421
Dental Education— " Harris.......... 425
To the Deans of the Dental Colleges in the United States of North America. Adolf Petermann.......... 427
The Care and Treatment of Exposed Pulps. B. F. Luckey.......... 430
Porcelain Facings for Carious Teeth. E. C. Moore.......... 433
About Plastic Stoppings. C. E. F.......... 435

REPORTS OF SOCIETY MEETINGS:

Illinois State Dental Society.......... 436
Southern Dental Association.......... 445
American Medical Association.......... 449
Chicago Dental Society.......... 455
Central Dental Association of Northern New Jersey.......... 458
New York Odontological Society.......... 461

CORRESPONDENCE:

Iodoform.......... 464
Marine Lint.......... 464
A Correction.......... 465

EDITORIAL:

The American Dental Association.......... 465
The Current Number.......... 467
Missing Numbers.......... 468
Our Book Table.......... 468

CURRENT NEWS AND OPINION:

A Fine Chance.......... 472
Pre-historic Decay of the Teeth.......... 472
The Saratoga Meeting.......... 473
Askings and Answers.......... 474

